# Sinonasal Lymphoma Presenting as a Probable Sanctuary Site for Relapsed B Acute Lymphoblastic Leukaemia: A Case Report and Review of the Literature

**DOI:** 10.1155/2015/697957

**Published:** 2015-11-30

**Authors:** W. Y. Lim, R. Care, M. Lau, S. Chiruka, P. J. D. Dawes

**Affiliations:** ^1^Dunedin School of Medicine, University of Otago, Dunedin, New Zealand; ^2^Department of ORL-HNS, Dunedin Hospital, Dunedin, New Zealand; ^3^Southern Community Laboratories, Dunedin, New Zealand; ^4^Department of Haematology, Dunedin Hospital, Dunedin, New Zealand

## Abstract

Sinonasal lymphoma is a non-Hodgkin lymphoma (NHL) representing 1.5% of all lymphomas. It presents as an unremitting ulceration with progressive destruction of midline sinonasal and surrounding structures. Poor prognosis warrants early treatment although diagnosis is challenging and frequently delayed. It is usually primary in origin and to our knowledge the sinonasal region has never been reported as a sanctuary site in leukaemia/lymphoma relapse. We present a unique case of B-cell ALL (acute lymphoblastic leukaemia) with late relapse to the nasal septum as a sinonasal lymphoblastic lymphoma and with genetic support for this as a sanctuary site.

## 1. Background

Sinonasal lymphoma presents as an unremitting ulceration affecting midline sinonasal region and surrounding structures [1, 2]. It is a form of non-Hodgkin lymphoma (NHL) comprising around 1.5% of all lymphomas [3–5].

A case of rapid destruction of the nose and face was first described by McBride in 1897 [6]. In 1933, Stewart [2] reported ten patients with chronic inflammatory processes involving midline nasal region and coined the term lethal granulomatous ulceration of the nose; by the 1950s the term “lethal midline granuloma” was in common use [7]. Friedmann (1955) reviewed the pathology and recognised two distinct pathologies: one is a generalised vascular, glomerular, and granulomatous lesion (Wegener's granulomatosis) and the other a more indolent granulomatous lesion [7]. Michaels and Gregory (1977) suggested that the cytological features of necrosis with atypical cellular exudate were consistent with histiocytic lymphoma [8]. Immunofluorescence studies by Ishii et al. subsequently demonstrated this to be a form of T-cell lymphoma [9]. Advances in immunohistochemical phenotyping resulted in subcategorization of sinonasal lymphoma according to its respective lineage origin, those of T-cell, NK/T-cell, nasal type (ENKL), and B-cell lymphomas [3, 4].

Sinonasal lymphoma is rare in Western countries with higher prevalence in South America and Asia [4]. Patients usually present in their fourth and fifth decades, with a male to female ratio of 2 : 1 [3, 10, 11]. Symptoms at presentation include nasal congestion, purulent nasal discharge, facial swelling, epistaxis, visual disturbance, and headache [3, 4, 10, 11]. Medical attention is often delayed for many months, partly due to the dismissal of urgency of symptoms [10–12] and coexistence/confusion with rhinosinusitis [11]. A wide range of differential diagnoses includes those of inflammatory, neoplastic, and infectious origins [4, 10, 11] and presence of both atypical and inflammatory lymphocytes in specimens may hinder accurate diagnoses [4, 11]. Five-year survival is poor, ranging from 24 to 65% [3, 13]. Prognosis is better in patients with an earlier Ann Arbor staging [14] at presentation, a smaller sized lesion, absence of B symptoms, nonelevated lactate dehydrogenase (LDH), absence of lymphadenopathy, and lymphomas of T-cell lineage [3, 4]. A study reporting 14 aggressive, large B-cell lymphomas of the paranasal sinuses suggested central nervous system (CNS) prophylaxis for aggressive lymphomas due to the high rate of CNS relapse [5].

We report a unique case of B-lymphoblastic lymphoma presenting as a late relapse of B-acute lymphoblastic leukaemia (B-ALL) in the sinonasal space which we believe represents a newly described sanctuary site.

## 2. Case Report

**Figure 1:**
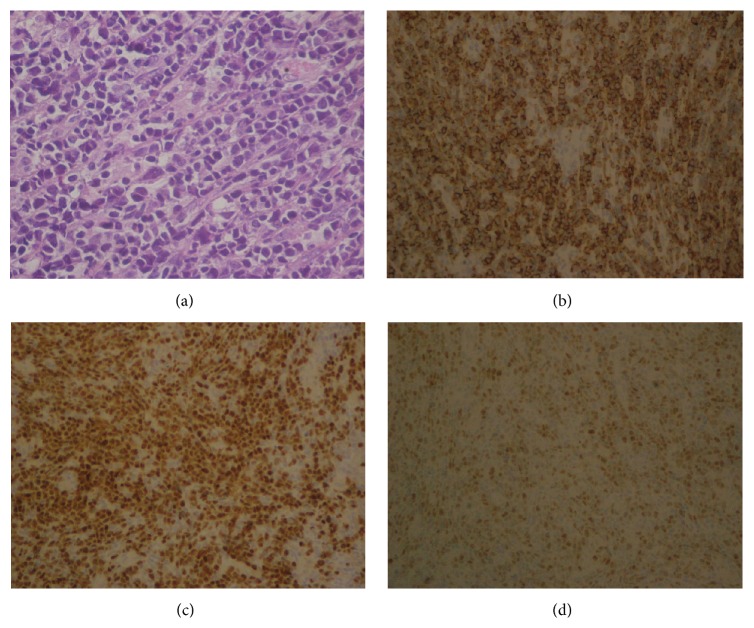
Histology of the nasal septal biopsy. (a) H&E High Power showing immature cells with hyperchromatic nuclei, occasional nucleoli, and high nuclear cytoplasmic ratios. (b) CD34 immunohistochemistry: positive membranous staining (CD34 marker of immature cells). (c) PAX-5 immunohistochemistry: positive nuclear staining (PAX-5 is a broad-spectrum B-lymphoid lineage marker). (d) Tdt immunohistochemistry: positive nuclear staining (Tdt marker of immature cells, favouring lymphoblastic lineage).

A 44-year-old Caucasian woman presented with a three-month history of nasal obstruction, yellow nasal discharge, and forehead pain. She was initially treated for allergic rhinitis and referred to the ENT department when symptoms persisted. Examination revealed a mass involving the nasal septum and the lower part of the nasal cavity and extending into the nasopharynx. Histology showed a monomorphic infiltrate of immature cells positive for CD19, PAX-5, HLADR, CD34, Tdt, and CD56 consistent with a diagnosis of B-cell ALL (Figure 1).

There was a history of B-ALL, with normal female karyotype at diagnosis, six years prior to current presentation. Due to poor risk features, including a high presenting white cell count of 122 × 10^9^/L, CNS disease at presentation, and presence of residual leukemic cells on flow cytometry after first cycle of induction, she had been treated with eight cycles of Hyper-CVAD followed by a cyclophosphamide/total body irradiation conditioned allogeneic stem cell transplant from a sex-mismatched, unrelated donor. She had suffered from hyperacute graft-versus-host disease (GVHD) which was steroid responsive and chronic GVHD of the liver treated with cyclosporine. This resolved and she had not been on any immunosuppression for 20 months at the time of presentation.

**Figure 2:**
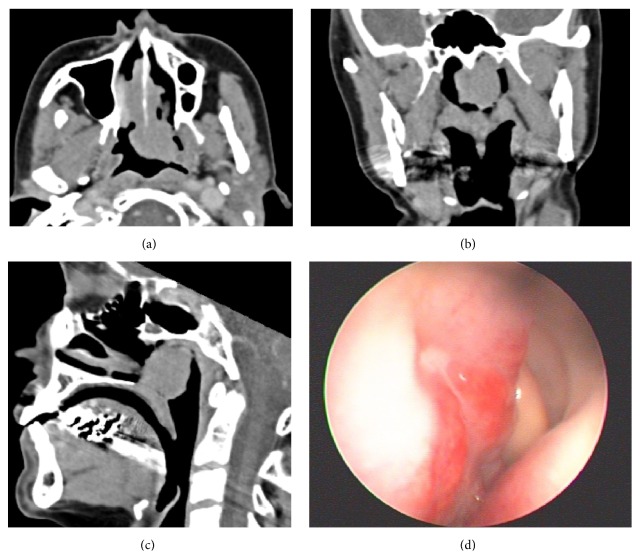
Imaging showing nasal septal lesion. CT images: (a) axial, (b) coronal, and (c) sagittal view. Endoscopic view left side: (d) lesion arising from the posterior part of the left nasal septum.

Pretreatment staging revealed an isolated sinonasal B-ALL, Ann Arbor stage 1 [14]. The LDH level was normal, CT demonstrated a nasal mass at the posterior septum extending into the nasopharynx (Figure 2), and there was no evidence of disease in the neck, chest, and abdomen. Bone marrow biopsies, CSF analysis by cytospin, and flow cytometry were normal.

Chimerism analysis by FISH revealed 100% XY cells in the bone marrow and peripheral blood. FISH analysis of the nasal biopsy showed most cells (191 out of 200) to have three copies of chromosome X with only 9 having chromosomes X and Y, consistent with relapse from initial female leukaemia cells. Further cytogenetic analysis of the sample was unsuccessful.

Induction chemotherapy was initiated according to the UKALL12 protocol. This was complicated by neutropenic fever, fatty infiltration of liver, hyponatraemia secondary to antibiotic induced SIADH, quite marked weight loss, and peripheral neuropathy secondary to vincristine. The L-asparaginase lowered her antithrombin levels and antithrombin concentrates were given to counteract her hypercoagulable state. Due to the significant toxicity chemotherapy was discontinued after Phase 1 induction and the planned CNS prophylaxis with high dose methotrexate was cancelled as she was too unwell with an ECOG performance status of three. She then received 3600 cGy consolidative radiotherapy in 20 fractions to the nasal cavity over four weeks. There was no evidence of systemic relapse at diagnosis or after completion of treatment.

Two months later she complained of hard crusts within her nose. Examination revealed a septal perforation with some necrotic bone at the posterior half of the nasal septum and associated crusting of the nasal cavity, colonised by pseudomonas. A persistent nonspecific opacity within the ethmoid and maxillary sinuses was also identified on CT imaging. The crusts were removed under local anaesthetic and necrotic bone was debrided. She was treated medically for suspected sinusitis secondary to local irradiation. Examination at her one-month and eleven-month follow-up revealed a septal perforation with healthy mucosal edges. There was no suggestion of recurrence of her sinonasal lymphoblastic lymphoma or leukaemia eighteen months after completion of chemoradiotherapy.

## 3. Discussion

B-cell lymphoblastic leukaemia/lymphoma (B-LBL/ALL) is a form of Precursor Lymphoid Neoplasm under the World Health Organization (WHO) classification of lymphoid neoplasm [15] constituting 2% of lymphoid malignancies [16].

LBL and ALL have similar clinical, cytogenetic, and histological features and are frequently treated as the same disease. Clinical distinction of LBL from ALL relies on the fact that LBL shows minimal involvement of blood or bone marrow [17]. They are grouped under the same entity as precursor lymphoblastic leukaemia/lymphoma as it was recognised that an initial leukemic picture could evolve to involve extramedullary lesions [18]. Less than 10% of LBL are of B-cell lineage, the rest being of T-cell lineage [19].

Extramedullary relapse in ALL is not uncommon but usually involves the lymph nodes, CNS, spleen, liver, and testis [20]. The development of sinonasal lymphoma in a paediatric patient with a past history of precursor B-ALL had been described but its immunophenotype was that of a diffuse large B-cell lymphoma, of lymphomatoid granulomatosis type [13].

The CNS and testes are well-recognised sanctuary sites for extramedullary relapse, protected from antileukemic T-lymphocytes and a graft-versus-leukemic effect possibly through biological barriers or increased blast tropism for these regions [20, 21]. These organs are prone to inadequate drug penetrance following intravascular administered chemotherapy [22]. We have found no reported case of the sinonasal region acting as a sanctuary site for ALL relapse.

Prognosis of adult ALL is poor with only 30–40% of patients achieving long-term disease remission [23]. A high white cell count at presentation increases the risk of relapse [24]. Our patient was given an induction phase of chemotherapy according to the UKALL12 protocol although this was not fully completed. The chemotherapy was poorly tolerated; hence intensification, consolidation, and maintenance chemotherapy was not planned. A second allogeneic stem cell transplant was not planned due to insufficient evidence supporting this treatment after GVHD especially given the 100% donor chimerism in the blood and bone marrow. The sinonasal space was considered a sanctuary site which shielded the leukemic cells from graft-versus-leukaemia effect; this is supported by the tumour showing female heritage and cytology consistent with her original B-ALL.

## 4. Conclusion

We report a sinonasal lymphoma as an isolated extramedullary relapse of B-ALL. This is an unusual site for B-ALL relapse and the female lineage of the tumour cells strongly supports our contention that the nasal septum represents a newly described sanctuary site for lymphoma.

Disease relapse is frequently diagnosed late further worsening an already grave prognosis of the disease. Early diagnosis improves clinical outcome highlighting the need for an awareness that patients presenting with sinonasal symptoms refractory to initial medical treatment are at risk of relapse.
